# COVID-19 outbreak prediction using Seq2Seq + Attention and Word2Vec keyword time series data

**DOI:** 10.1371/journal.pone.0284298

**Published:** 2023-04-26

**Authors:** Yeongha Kim, Chang-Reung Park, Jae-Pyoung Ahn, Beakcheol Jang

**Affiliations:** 1 Department of Computer Science, Sangmyung University, Seoul, South Korea; 2 Technological Convergence Center, Korea Institute of Science and Technology, Seoul, South Korea; 3 Graduate School of Information, Yonsei University, Seoul, South Korea; 4 Research Resources Division, Korea Institute of Science and Technology, Seoul, South Korea; Yeungnam University, REPUBLIC OF KOREA

## Abstract

As of 2022, COVID-19, first reported in Wuhan, China, in November 2019, has become a worldwide epidemic, causing numerous infections and casualties and enormous social and economic damage. To mitigate its impact, various COVID-19 prediction studies have emerged, most of them using mathematical models and artificial intelligence for prediction. However, the problem with these models is that their prediction accuracy is considerably reduced when the duration of the COVID-19 outbreak is short. In this paper, we propose a new prediction method combining Word2Vec and the existing long short-term memory and Seq2Seq + Attention model. We compare the prediction error of the existing and proposed models with the COVID-19 prediction results reported from five US states: California, Texas, Florida, New York, and Illinois. The results of the experiment show that the proposed model combining Word2Vec and the existing long short-term memory and Seq2Seq + Attention achieves better prediction results and lower errors than the existing long short-term memory and Seq2Seq + Attention models. In experiments, the Pearson correlation coefficient increased by 0.05 to 0.21 and the RMSE decreased by 0.03 to 0.08 compared to the existing method.

## Introduction

Coronavirus disease (COVID-19), which was first reported in Wuhan, China, in November 2019, is a worldwide epidemic, which has caused numerous infections and deaths, and the social damage is also very large. As of 2022, vaccines and treatments have been developed to prevent COVID-19. However, despite the high vaccination rate worldwide, the number of infections and deaths continues to increase. To reduce this damage, various studies have been conducted to predict COVID-19 [1–5].

Most of these studies used mathematical prediction models, such as susceptible-infectious-recovered (SIR) and susceptible-exposed-infectious-removed (SEIR); however, oversimplified models and predictions based on incorrect assumptions have an adverse effect on the long-term prediction of COVID-19 [6]. Thus, with the recent development of artificial intelligence technology and the rapid development of computer hardware performance, research using artificial intelligence to predict COVID-19 has emerged.

For example, [7] introduced a model combining an SEIR-based epidemiologic model and an artificial neural network to predict the number of confirmed COVID-19 cases in Bangladesh. [8] used long short-term memory (LSTM) recurrent neural networks to predict recovered COVID-19 cases in the United States, India, and Italy for seven days. [9] used recurrent neural network (RNN)-based LSTM models, including deep LSTM, convolutional LSTM, and bidirectional LSTM, to detect the coronavirus disease in 32 states and federal regions of India. [10] calculated the number of confirmed COVID-19 cases in five countries (USA, India, Brazil, Russia, and France) using artificial neural network (ANN) and RNN-based LSTM models and conducted a comparative study of the model performances. In [11], three neural network models, namely, multi-head attention, LSTM, and convolutional neural network (CNN), and a Bayesian optimization technique, an algorithm that finds the optimal hyperparameters for each model, were used together to predict the occurrence of COVID-19.

However, in COVID-19 prediction studies that use deep learning and artificial intelligence, the prediction accuracy is low due to the lack of training data when the outbreak period of COVID-19 is short. Most deep learning-based COVID-19 prediction studies [8–14] were published in 2020. However, at the time, there were not enough data to train the model because the number of cases was small, just after the outbreak of COVID-19. In this paper, we propose an improved prediction model with low learning error and high accuracy even when the period of outbreak of an epidemic is small by using existing epidemic prediction models, such as LSTM and CNN, which can be used to predict not only COVID-19 but also other infectious diseases.

## Related works

Several recent studies have predicted the damage caused by COVID-19. They have largely focused on mathematical prediction models (e.g., SIR and SEIR) [1–6] and prediction methods using deep learning and machine learning techniques [8–14] some studies used both methods.

## Mathematical prediction models

Based on the SIR model [1], demonstrated a time-dependent transition from the outbreak of COVID-19 in China by estimating three variables: failure rate, time-dependent recovery rate, and time-dependent mortality rate. They analyzed COVID-19 epidemiological data in Iran from January 22 to March 24, 2020 and investigated and predicted results until April 15, 2020. [2] reported a COVID-19 outbreak in India using existing COVID-19 epidemiological data using the mathematical model SIR(D) for prediction. According to the prediction results of the model, if the number of people infected with COVID-19 increased after May 3, 2020, it will resume at the end of June, and the peak of the epidemic is predicted for the first week of July. Furthermore, [3] collected time-series data of COVID-19 in Italy from January 22, 2020, to April 2, 2020, and predicted the outbreak of COVID-19 using an infectious disease dynamic extended-susceptibility-removal model. This forecast model predicted a total of 182,051 infections due to the current national lockdown, which will end at the end of August.

[4] introduced a time-window mechanism for dynamic data analysis and proposed a time-window-SIR model for predicting epidemic outbreaks using machine learning methods. From February to July 2020, they collected and analyzed COVID-19 data from seven countries: China, Korea, Italy, Spain, Brazil, Germany, and France. [5] studied cumulative and daily coronavirus case data of COVID-19 outbreaks in China. By plotting the number of cases against the distance from the epicenter of the COVID-19 outbreak in China and Italy, the SIR model reproduced the data for China with high accuracy for a given parameter value and predicted when the epidemic could be expected to end.

In [6], used "Logistic growth curve model", "SIR model", "Time Interrupted Regression model", etc. to predict the maximum number of cases for regions with high COVID-19 outbreaks in India, and a 3-week quarantine period Assess the impact on the outbreak of COVID-19.

### Limitations of mathematical models

In [7] the SIR model was built using data from the first three months of the COVID-19 epidemic in Isfahan, Iran, and the problems with the SIR model were analyzed. In the experimental results of several other studies, it was found that the prediction results of the SIR-based models for COVID-19 do not match the actual data in the long-term prediction and that they are oversimplified and thus do not predict the COVID-19 pandemic. The main problems were that the developed models had a great influence on the course of the disease, and that SIR modeling is based on assumptions that are not necessarily true.

### Machine learning and deep learning prediction methods

In [8], introduced a SEIR-based integrated neural network model to predict the number of COVID-19 cases in Bangladesh. This model was created by combining a SEIR dynamics model with an ANN. The model was trained using 250 days of training data. The experimental results showed that the prediction accuracy for a COVID-19 outbreak was between 90% and 99%. [9] presents a comparative analysis of machine learning and soft computing models to predict COVID-19 outbreaks as an alternative to the SEIR model. Among the several models investigated, multilayer perceptron (MLP) and adaptive network-based fuzzy inference systems (ANFIS) showed the best results in COVID-19 prediction.

[10] uses the support-vector-machine (SVM) model to predict infection of COVID-19 in real time. [10] investigated COVID-19 confirmed, death and recovery case predictions, and collected data such as death, recovery, and location data (latitude and longitude) from COVID-19 worldwide from January 22, 2020 to April 25, 2020.

[11] predicted the COVID-19 recovery rate in the US using existing mathematical models SEIR, SIR, SIRQ, ARIMA, ARMA, and deep learning-based prediction model LSTM. As a result of the experiment, when predicting the recovery rate of covid-19 one week later, the LSTM model showed the best performance with a 3% MAPE error.

In [12], used LSTM RNNs to predict COVID-19 recovery cases in the United States, India, and Italy for seven days. The performance of the model was verified by calculating the mean absolute percentage error for the actual recovery cases and predicted results. According to the experiments, the LSTM model yielded accurate results, with a minimum error not exceeding 3%. used RNN-based LSTM models including deep LSTM, convolutional LSTM, and bidirectional LSTM to predict the number of COVID-19 cases. The experiment showed high accuracy for short-term prediction, with an error of less than 3% for daily prediction and less than 8% for weekly prediction.

In [13], the number of COVID-19 cases in the five countries most affected by COVID-19 in the world (USA, India, Brazil, Russia, and France) was calculated using an ANN and an RNN. A comparative study was conducted on the performance of each model using models based on LSTM. The experimental results indicated that the LSTM model outperformed the ANN model. In [14], three neural network models, namely, multi-head attention, LSTM, and CNN, and a Bayesian optimization technique, an algorithm that finds the optimal hyperparameters in each model, were used together to predict the occurrence of COVID-19. The proposed method showed better performance in terms of long-term and short-term predictions compared to the existing method.

### Comparison of related works with our research

Table 1 compares our model with the previously investigated study. [1–6] in Table 1 is a study derived from the SIR model, all mathematical models for predicting the existing COVID-19. Because these models basically predict COVID-1 based on CDC data, the data is updated on a daily or weekly basis, which is disadvantageous for real-time prediction. In addition, since the number of infections is small in the early stages of the COVID-19 outbreak, the prediction results vary significantly by each parameter entering the model, and it is difficult to find appropriate parameters because there is not enough data to consider several environmental variables.

**Table 1:** Compare related work with our research.

Mathematical prediction models			
**Prediction model**	**Used data**	**Available real-time prediction**	**Predictive performance when data sets are small**
[1] SIR + GAM	CDC	False	Bad
[2] SIRD	CDC	False	Bad
[3] Extended SIR	CDC	False	Bad
[4] TW-SIR	CDC	False	Bad
[5] SIR	CDC	False	Bad
[6] Logistic growth curve model, SIR, etc.	CDC	False	Bad
**Machine Learning and deep learning prediction methods**			
**Prediction model**	**Used data**	**Available real-time prediction**	**Predictive performance when data sets are small**
[8] SEIR + ANN	CDC	False	Bad
[9] SEIR + MLP, ANFIS	CDC	False	Bad
[10] SVM	CDC	False	Bad
[11] LSTM,	CDC	True	Bad
[12] LSTM, Bi-LSTM, Conv-LSTM	CDC	True	Bad
[13] LSTM	CDC	True	Bad
[14] multi-head attention, LSTM, and CNN	CDC	True	Bad
Proposed model	CDC, News Google trend	True	Good

[8–14] of Table 1 are studies that predict COVID-19 by converging SIR and deep learning technology or using only deep learning technology. [8,9] of Table 1 combine the traditional mathematical model, SEIR, with artificial intelligence or deep learning techniques to find the appropriate parameters required for the SEIR model. If the data is not sufficient, there is a possibility of choosing the wrong parameter. [10] of Table 1 proposes a prediction method using the SVM machine learning technology. This study also relies solely on CDC data, so it is not suitable for real-time prediction, and may be suitable for short-term prediction if there is little data for model learning, but time series prediction for longer than a week is somewhat bad compared to the accuracy of the LSTM model. [12–14] in Table 1 propose several deep learning-based models such as LSTM, and Conv-LSTM, Bi-LSTM, Multi-head attention LSTM, and CNN. These studies may be suitable for long-term predictions, but with a similar drawback to the mathematical models described earlier, learning data can lead to overfitting, which still does not solve the problem of prediction accuracy when data is small. Our model, the last part of Table 1, is advantageous for real-time prediction because it uses web data as well as CDC data, and even in the early stages of COVID-19, multiple web data related to COVID-19 can be used together to alleviate the overfitting problem.

## Methods

### Data collection method

To predict the COVID-19 outbreak in the United States in 2020, from April 5, 2020, to June 27, 2021, data from the five states with the highest number of COVID-19 outbreaks (California, Texas, Florida, New York, and Illinois) were used. We collected 49,456 New York Times news articles written between April 5 and June 27, 2021, as well as data on the weekly incidence of COVID-19 in the United States. The collected news articles used the API provided by The New York Times, and COVID-19 data by country, officially provided by the World Health Organization, were used as COVID-19 data.

### Finding words related to COVID-19 using the Word2Vec model

The Word2Vec model was trained with the collected New York Times news articles to extract several words related to COVID-19. Here, the Word2Vec model encoded a word as an N-dimensional vector, which addressed a limitation of one-hot encoding that it cannot apply significant similarity between word vectors. The Word2Vec model is a neural network model that divides a sentence into words, the minimum unit of a sentence, and encodes each word into a word vector space by learning the relationship between each word and its surrounding words [15]. The greater the significant correlation between each word pair, the closer the distance between the vectors of the corresponding words. There are two major learning methods for the Word2Vec model, the continuous bag of words and skip-gram; in this study, the skip-gram method was used. For hyperparameters, a vector size of 100 dimensions, a window size of 5, and a minimum number of word mentions of 5 were used.

Here, the vector size determines the number of dimensions of the vector used to encode a word, and the window size represents the maximum number of adjacent words that the Word2Vec model used for determining the similarity between words. The minimum number of word mentions means that only words with a minimum occurrence frequency of five or more were used the model. During preprocessing, each sentence was divided into words using the natural language processing package NLTK, and special characters, stopwords such as “at,” “it,” “on,” and “the,” and numbers were removed; then, the model was trained. Subsequently, using the trained Word2Vec model, 50 words that were closest to “covid19” and the word vector were selected, and data on the Google search rate of the word for each state in the United States were collected from Google Trends.

### Filtering words required for learning

The Pearson correlation coefficient is close to 1 when the two variables are positively correlated. Conversely, if there is a negative correlation, the coefficient is close to -1. However, if there is no significant correlation between the two variables, the coefficient approaches 0. The formula for calculating the Pearson correlation coefficient for two variables X = [$x_{1},x_{2},x_{3}$,…], y = [$y_{1},y_{2},y_{3}$,…] as follows [16].


$$P(x,y)=\frac{\sum_{i}^{n}\left(x_{i}-\bar{x})(y_{i}-\bar{y}\right)}{\sqrt{{\left(x_{i}-\bar{x}\right)}^{2}}\sqrt{{\left(y_{i}-\bar{y}\right)}^{2}}}$$ (1)

The two collected datasets, COVID-19 outbreak data and Word2Vec Google Trends time-series data, were normalized to values between 0 and 1, and Google keyword data with a Pearson correlation coefficient greater than or equal to 0.3 between the two datasets were selected. The root mean square error (RMSE) was used to sort them in descending order. The model then selected the five Google Trends time-series data with the lowest RMSE and used them to predict COVID-19.

RMSE is an indicator of the difference between the expected value and the actual value. RMSE was used to train the model using real data and data with the lowest error. The formula for calculating the RMSE for two variables X = [$x_{1},x_{2},x_{3}$,…], y = [$y_{1},y_{2},y_{3}$,…] as follow: [17]

$$R(x,y)=\sqrt{\frac{1}{n}\sum_{k}^{n}{\left(x_{k}-y_{k}\right)}^{2}}$$ (2)

**Fig 1:**
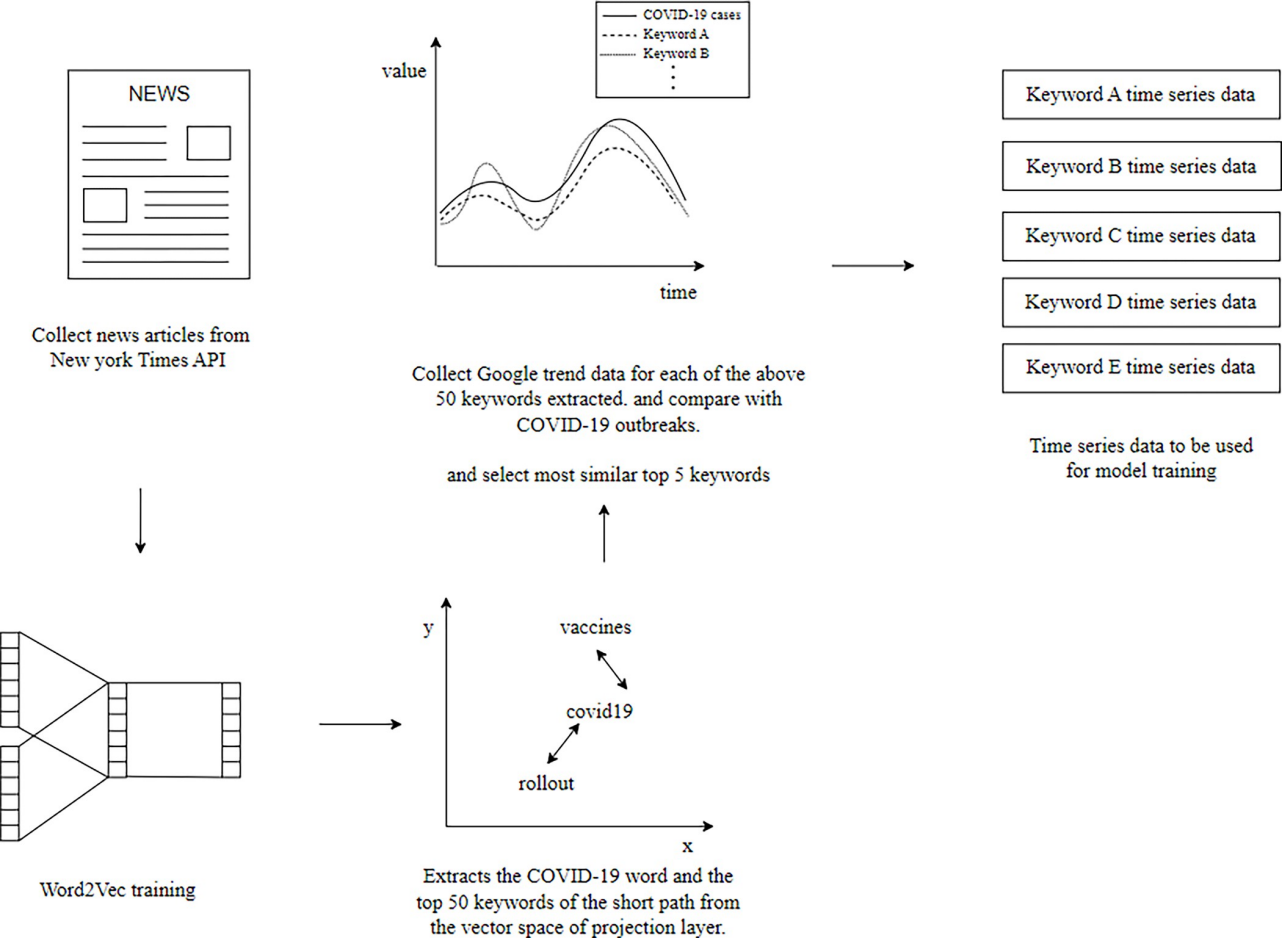
Extraction methodology of time series data for training the model.

Setting the Pearson correlation coefficient of the selected data as greater than 0.3 was based on the guidelines for the Pearson correlation coefficient [16]. Keywords, Pearson correlation coefficients, and RMSE for learning COVID-19 data for each state in the United States are listed in Table 2. Fig 1 presents a simplified figure of all the processes of extracting time series data for training the model through Word2Vec.

**Table 2:** Google trends keywords used to train the model.

States in the USA				
**California**	**Texas**	**Florida**	**New York**	**Illinois**
**(Keyword, PCC, RMSE)**	**(Keyword, PCC, RMSE)**	**(Keyword, PCC, RMSE)**	**(Keyword, PCC, RMSE)**	**(Keyword, PCC, RMSE)**
rollout, 0.62, 0.20	rollout, 0.59, 0.22	rollout, 0.60, 0.22	rollout, 0.82, 0.17	biontech, 0.69, 0.17
biontech, 0.38, 0.25	biontech, 0.46, 0.24	appointments, 0.53, 0.23	vaccines, 0.72, 0.20	illness, 0.35, 0.24
symptoms, 0.49, 0.30	vaccines, 0.41, 0.28	biontech, 0.53, 0.24	moderna, 0.62, 0.23	rollout, 0.31, 0.26
oxygen, 0.49, 0.36	antibodies, 0.40, 0.31	vaccines, 0.55, 0.25	symptoms, 0.50, 0.23	symptoms, 0.49, 0.27
vaccines, 0.30, 0.36	oxygen, 0.39, 0.34	moderna, 0.42, 0.27	polio, 0.54, 0.23	polio, 0.31, 0.27

### Training and test data

We used the COVID-19 and Word2Vec Google Trends time-series data collected between April 5, 2020, and June 27, 2021, as training data, with 10% used for validation. On July 1, 2021, COVID-19 data from November 30, 2021, and Word2Vec Google Trends time-series data were additionally collected and used as test data to test the model’s prediction results.

### Data preprocessing

**Fig 2:**
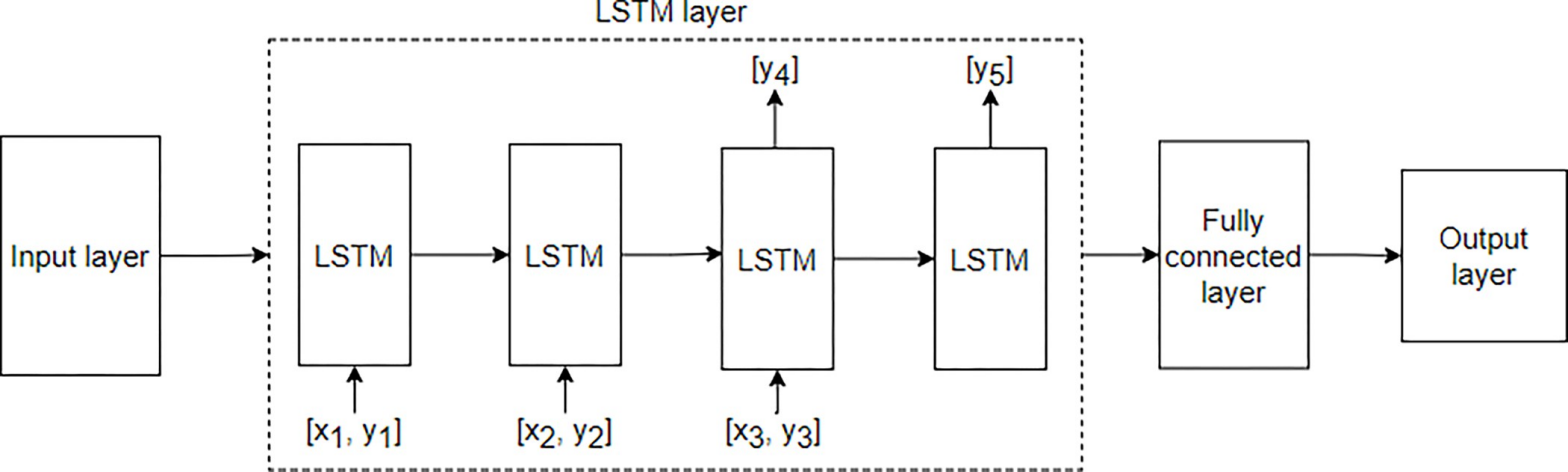
LSTM model structure.

**Fig 3:**
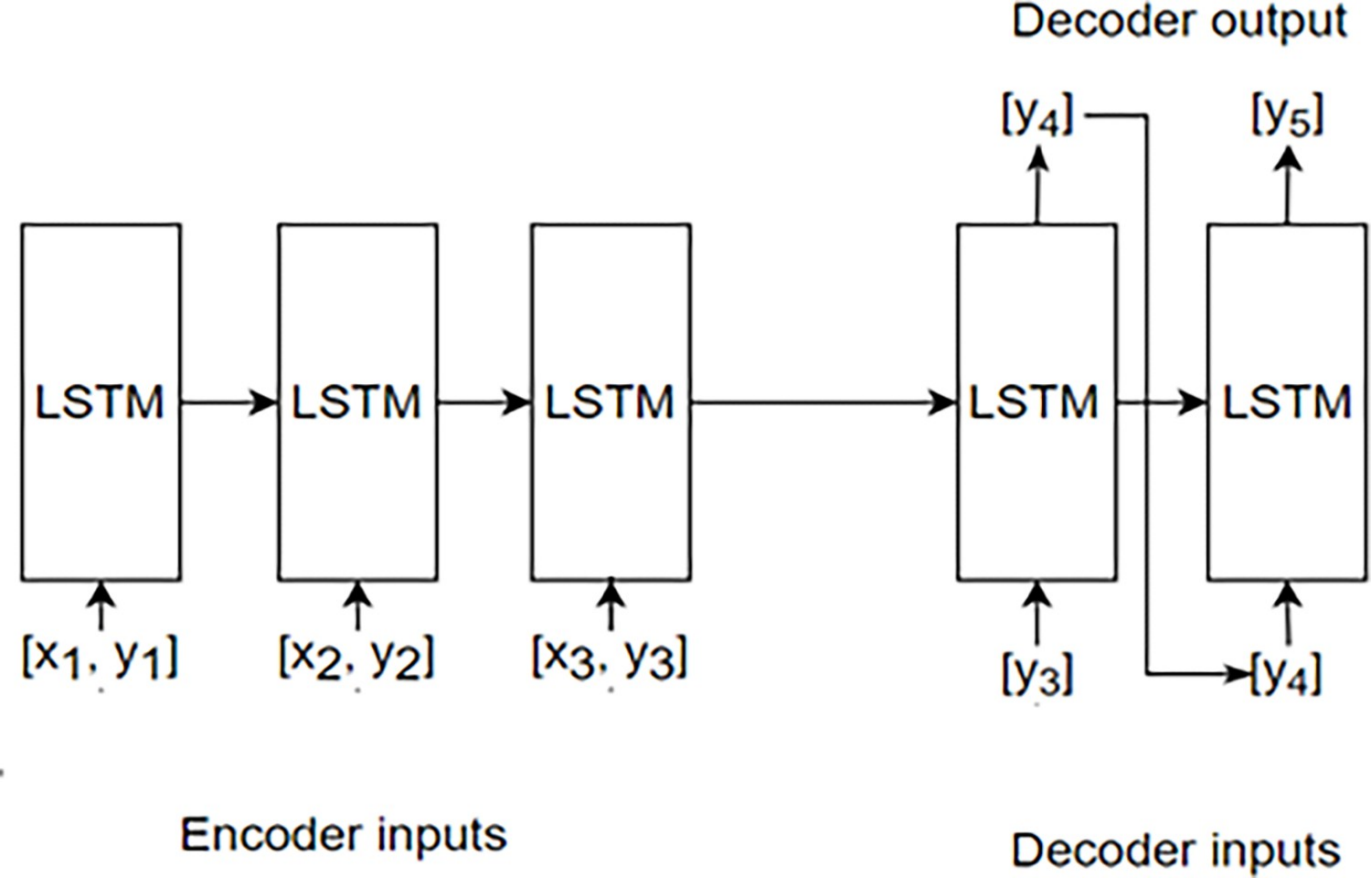
Seq2Seq model structure.

Preprocessing of the training, validation, and test data is required prior to training the model. Both, the Google Trends data to be used for the model, listed in Table 2, and the COVID-19 data for each state in the United States were normalized to a value between 0 and 1. Because the dataset for the prediction of the LSTM model should include temporal data as one-dimensional sequence data, the input data were converted into three-dimensional data of batch size, input sequence length, and number of input data points. For example, if the input data are [1, 2, 3,…10], the prediction data are [11, 12, 13,…20], the input sequence is 2, the sequence to be predicted is 1, and the input data. When the number of is 2, data are created in the following form: x = [[[1, 11], [2, 12]], [[2, 12], [3, 13]], [[3, 13], [4, 14]]…] y = [[13], [14], [15],…], where x is the data input to the model and y is the data to be predicted. The manner in which the data enter the model is briefly illustrated in Fig 2. In the case of the Seq2Seq model, three types of data were entered to train the model: encoder input, decoder input, and decoder output. When the input sequence is 2, the sequence to be predicted is 2, and the number of input data is 2, the encoder input is [[[1, 11], [2, 12]], [[2, 12], [3,13]]], [[3, 13], [4, 14]]…], the decoder input is [[11, 12], [12, 13], [13, 14],…] and the decoder output is [[12, 13], [13, 14], [14, 15],…]. The manner in which the data enter the model is briefly illustrated in Fig 3.

In the experiment, the past COVID-19 data and Google Trends data shown in Table 2 were entered as input data, and the data to be predicted became the future COVID-19 data.

### Model descriptions, hyperparameters, and prediction methods

First, the LSTM neural network (Fig 2) is a modification of an existing RNN. RNNs have the problem of vanishing gradient, in which the learning gradient does not decrease well when the input data become long.

To address this problem, memory, forget, and output gates are added inside the RNN cell. This determines the amount of past data to remember and the extent to which the current data should be reflected and passes the information to the next LSTM cell. Unlike a conventional RNN cell, it additionally passes the cell state so that the LSTM can process a longer sequence of inputs than the RNN. Seq2Seq (Fig 3) is a neural network that originally appeared for machine translation in natural language processing; it outputs a sequence of outputs different from the input sequence. The Seq2Seq model can be used not only for machine translation but also for time-series prediction.

**Fig 4:**
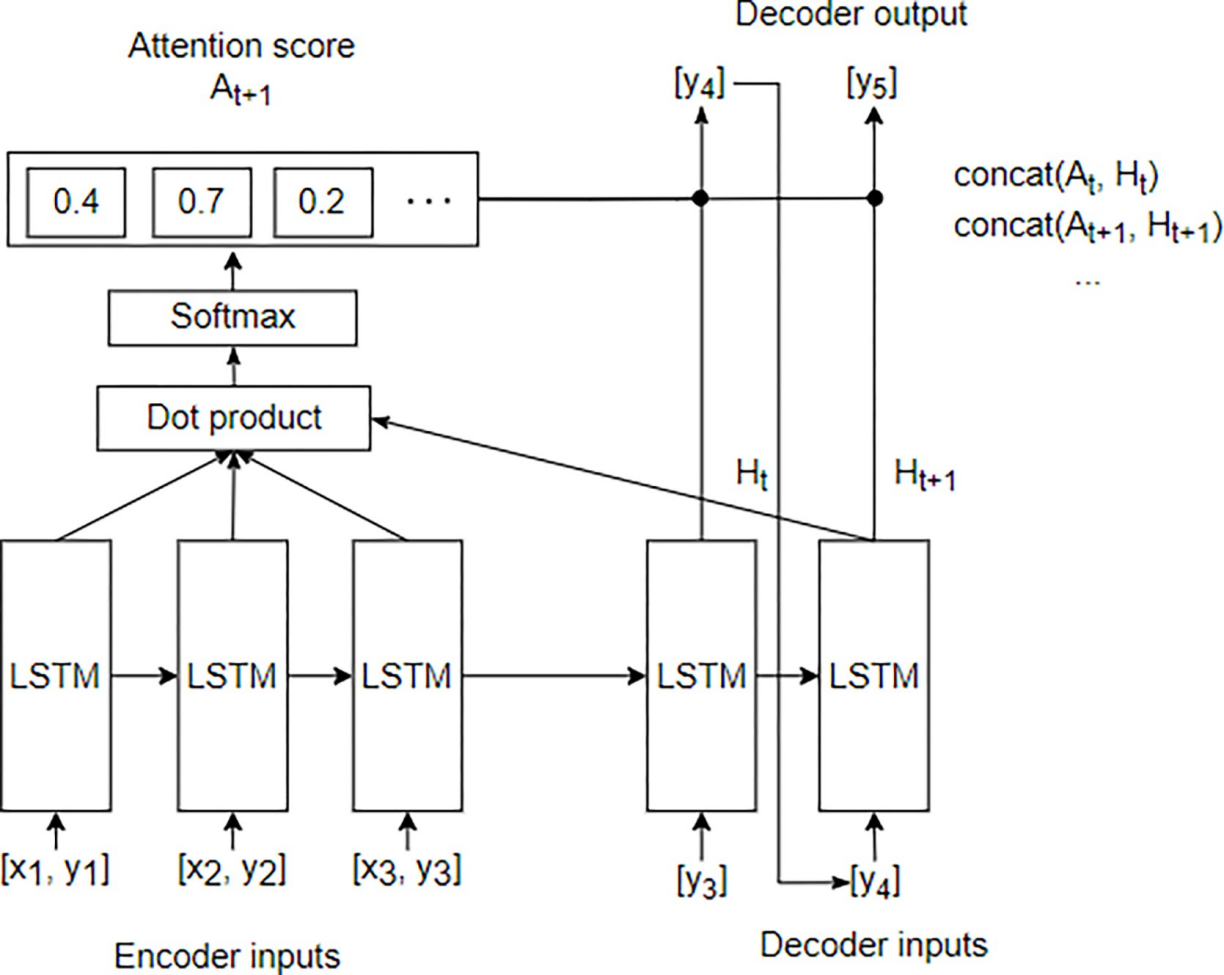
Seq2Seq model structure.

In [18], used several RNN-based time-series prediction models, such as bidirectional LSTM, convolutional LSTM, LSTM autoencoder, Seq2Seq, and Seq2Seq Attention, for long-term and short-term predictions. When various requirements were evaluated, the Seq2Seq model showed good performance in long-term and short-term predictions in time-series prediction tasks. Seq2Seq consists of an encoder and decoder composed of LSTM cells. When a sequence is input in the encoder, the encoder sends out the hidden state of the last LSTM cell of the encoder, that is, a context vector, which contains compressed information about the input data. The decoder predicts the next sequence based on the context vector output from the encoder, and the predicted sequence returns to the decoder input, and the next sequence is sequentially predicted [19]. When the input sequence of the existing Seq2Seq model becomes long, a bottleneck occurs as all the encoder information is compressed into one fixed vector, which adversely affects the prediction results. This problem can be solved using Seq2Seq + Attention (Fig 4). Conceptually, when the decoder sees the context vector, it refers to all hidden states of the encoder, and it is predicted by referring to which hidden state of the encoder to focus on at the current time step of the decoder. At this time, the decoder refers to the dot product of the hidden state in one LSTM cell of the decoder and all the hidden states of the encoder, put it in the softmax function, and output it. This output, which is called the attention score [20] and is expressed as a value between 0 and 1, indicates the importance of the hidden state of the encoder, to which the decoder should refer. There are several methods to determine the attention score, and we used the “dot product attention,” which has a simple implementation.

**Fig 5:**
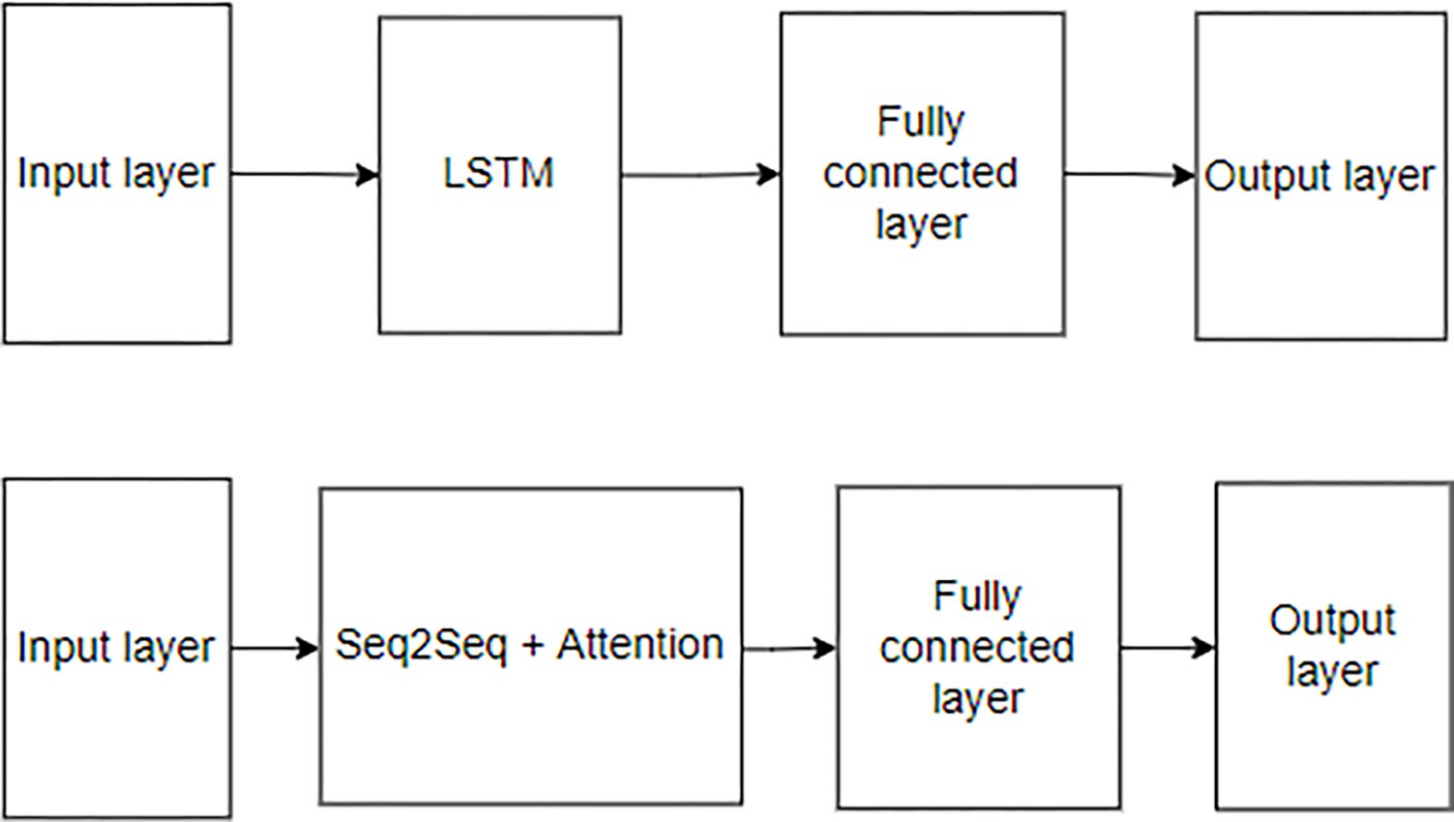
LSTM, Seq2Seq + Attention model structure (LSTM: Top, Seq2Seq + Attention: Bottom).

Fig 5 shows the structures of the existing LSTM and Seq2Seq + Attention models used in the experiment. Both models have structures that lead to an output layer through an input layer, LSTM or Seq2Seq + Attention, and a fully connected layer. Each LSTM and Seq2Seq cell consist of several LSTM cells, and each cell learns the time-sequence data for the input data. The internal structure of the LSTM and Seq2Seq + Attention is shown in Figs 2 and 3.

**Fig 6:**
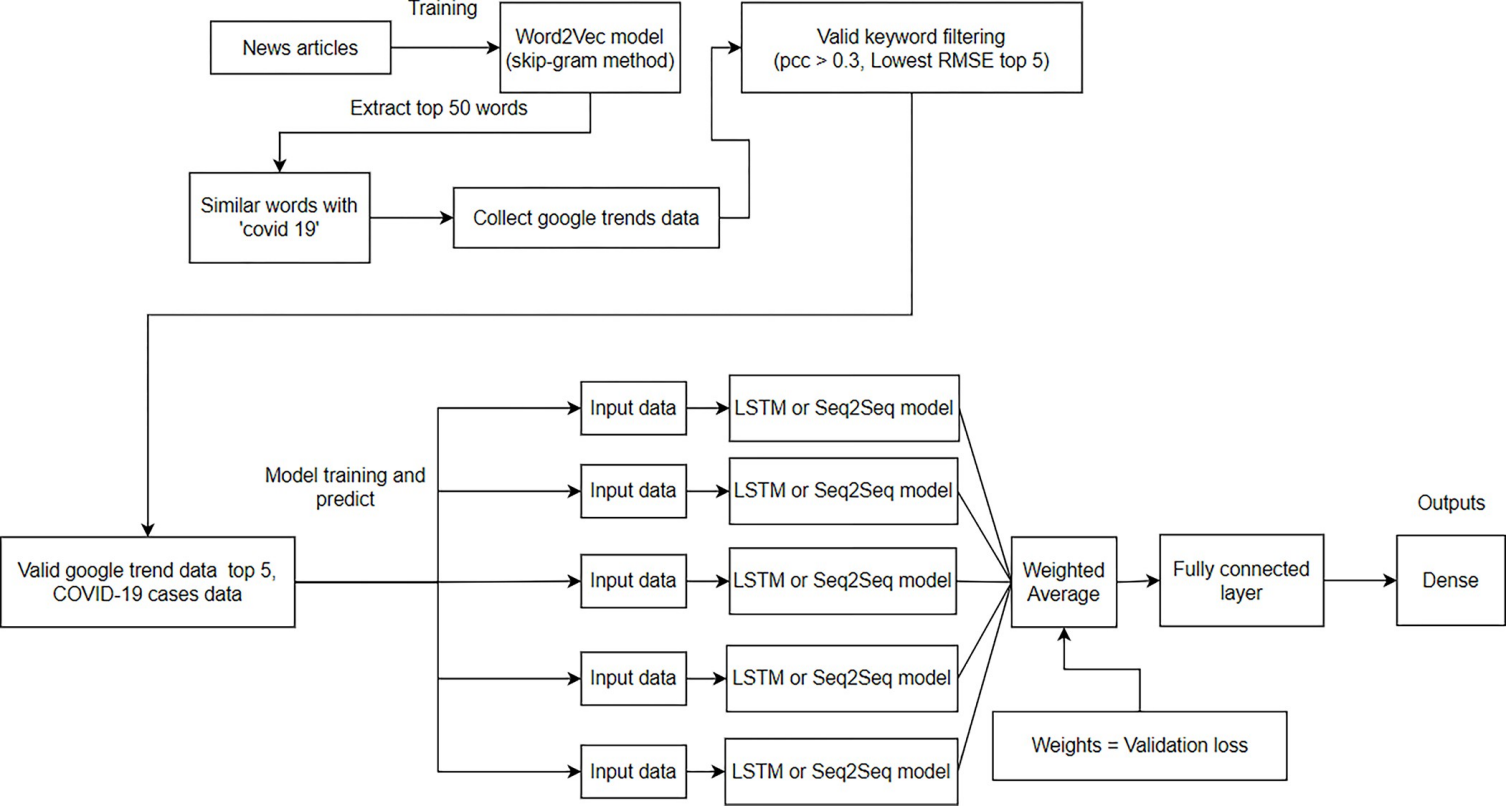
Proposed model architecture.

Fig 6 shows the overall structure of the proposed model (Word2Vec + LSTM/Seq2Seq + Attention). The Word2Vec was trained on The New York Times news data in advance, and N keywords related to “covid19” were extracted from the trained Word2Vec model (we set N = 50). We then collected Google Trends data from each of the 5 states for the 50 keywords. Then, to select data suitable for learning, the Pearson correlation coefficient and RMSE between the COVID-19 outbreak data and Google Trends data were compared. In this study, the five Google Trends data instances with the lowest RMSE among the data with a Pearson correlation coefficient of 0.3 or higher were selected as suitable data for learning (Table 1). After that, the finally selected Google Trends data (5 instances), past Google Trends data, and past COVID-19 data were input for each keyword through the LSTM/Seq2Seq + Attention model, and future COVID-19 data were predicted by each cluster model.

**Fig 7:**
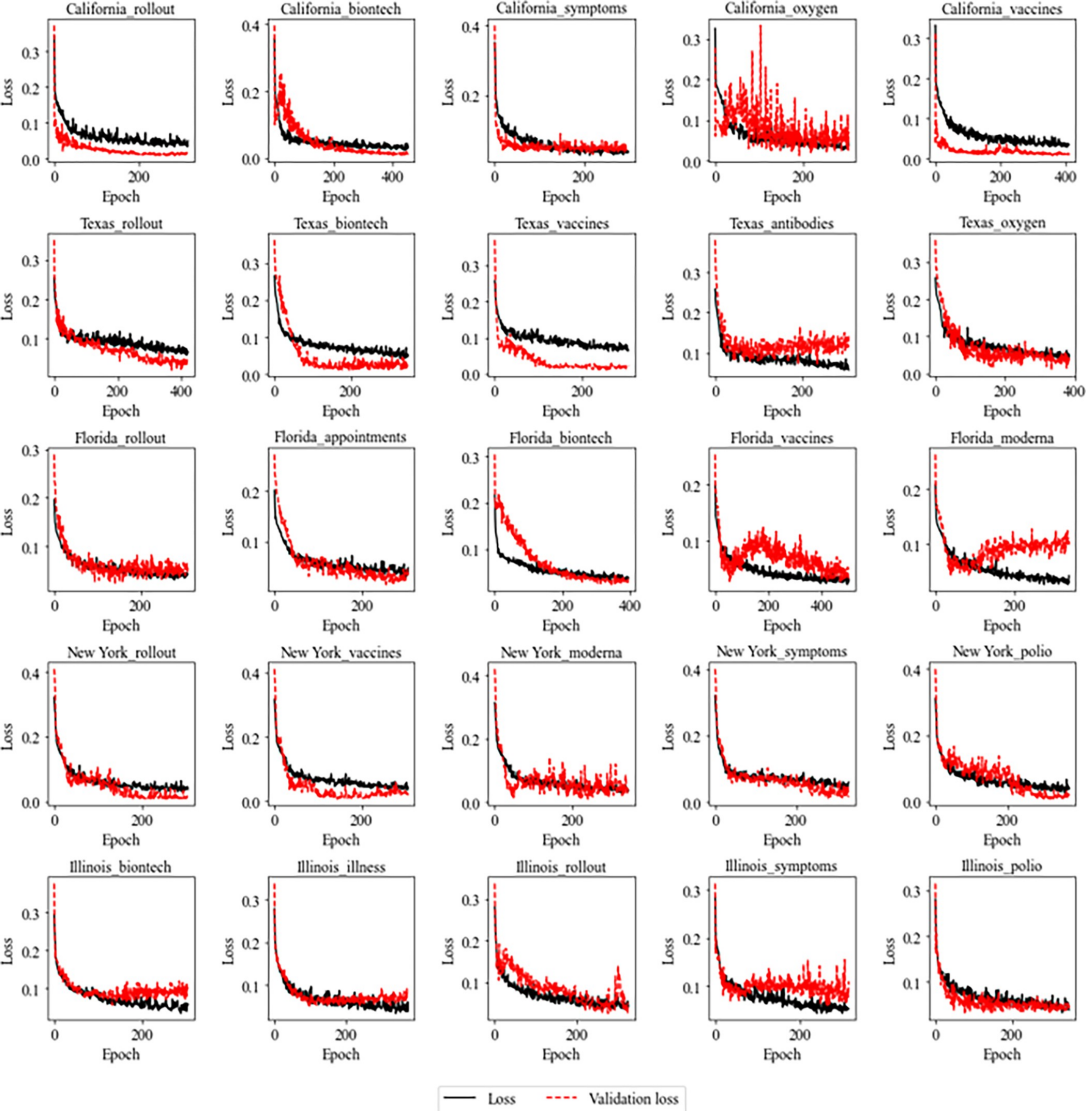
Learning error and verification data error according to the number of model trainings (LSTM).

**Fig 8:**
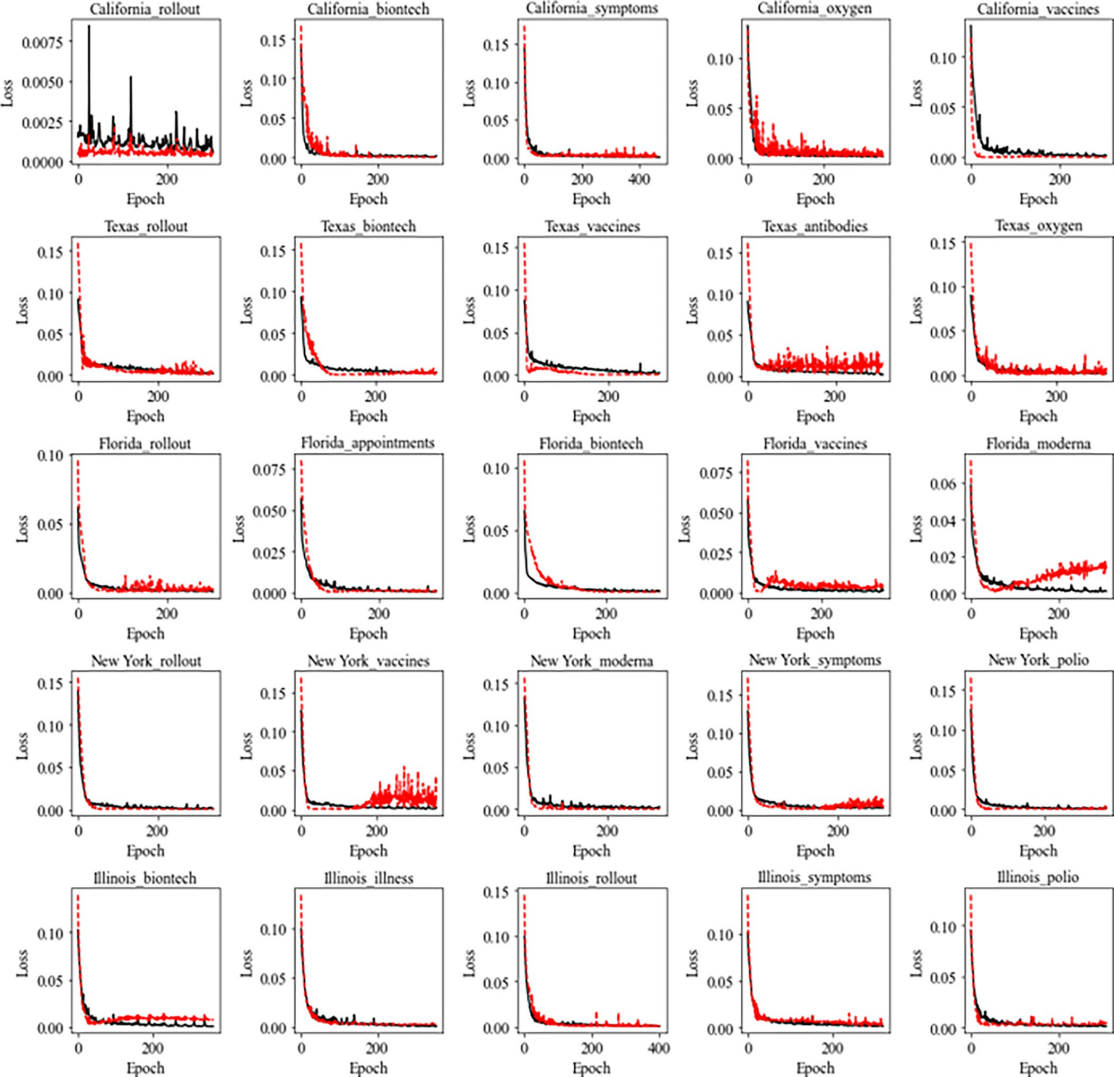
Learning error and verification data error according to the number of model trainings (Seq2Seq + Attention).

Figs 7 and 8 present the training records of each of the five cluster models trained with the Google Trends keyword time series data selected above. The losses of Figs 7 and 8 are the results of MAE’s calculations for the values the model predicted the training data, and the verification losses in Figs 7 and 8 are the calculations of MAE for the values predicted for the validation data. As shown in Figs 7 and 8, training errors continue to decrease as learning progresses, but in the case of verification errors, they decrease simultaneously with training errors, but at some point, the errors increase rapidly. To avoid overfitting the model, we stopped training when the loss on the validation data continues to increase and saved the weight with the smallest training error in the validation data.

Then, when the prediction results of the five models were weighted, averaged, and combined, the training error on the validation data was used as weight. Because the training error used the mean absolute error, the smaller the error between the actual and predicted values for the validation data, the smaller the training error. Therefore, Eq (3) was applied to the learning error E to reflect the prediction result with a small learning error.


$$\bar{E}=1-Norm(E,0,1)$$ (3)

Eq (3) normalizes the training error E on the validation data to a value between 0 and 1, and then takes the inverse of the value. Subsequently, using the prediction results predicted by each of the five models as weights, the weighted average is output through the fully connected layer.

The models compared in this experiment were as follows. The first method (existing method) collected time-series data for the keyword “covid19” by state from Google Trends and predicted the future data with LSTM and Seq2Seq + Attention models. The second method extracted keywords related to “covid19” from a pre-trained Word2Vec model, selected valid data for training, and made predictions using multiple LSTMs and Seq2Seq + Attention models. We used the two models to predict future COVID-19 in five US states and compared their performances.

The hyperparameters of the models are shown in Table 3. The input data were data from the past seven weeks to predict the COVID-19 outbreak after one to three weeks (target length). Each model was trained at 250–1200 times, and the loss on validation data was calculated each time the model was trained. After training, the weight with the least loss on the validation data was loaded to prevent overfitting of the model. While the model was being trained, if the model did not show better predictions than the existing validation data loss within 50 (patience), we terminated the training early. Dropout avoids overfitting by omitting parts of the neural network when training the model. Dropout was applied inside the LSTM cell. Unlike in general neural network models, dropout in RNN-based neural networks does not apply dropout to past information of previous LSTM cells but applies it to input data at the current time step [20].

**Table 3:** Hyperparameter of model.

Parameter name	Prediction method			
	LSTM		Seq2Seq + Attention	
	normal	proposed	normal	proposed
LSTM units	32	32	-	-
Encoder units	-	-	32	32
Decoder units	-	-	32	32
FC Layer units	16	16	16	16
Learning rate	0.001	0.001	0.001	0.001
Input length	7 weeks	7 weeks	7 weeks	7 weeks
Target length	3 weeks	3 weeks	3 weeks	3 weeks
Min epochs	250	250	250	250
Max epochs	1200	1200	1200	1200
Patience	50	50	50	50
Dropout	0.1	0.1	0.1	0.1

### Experimental results and analysis

**Fig 9:**
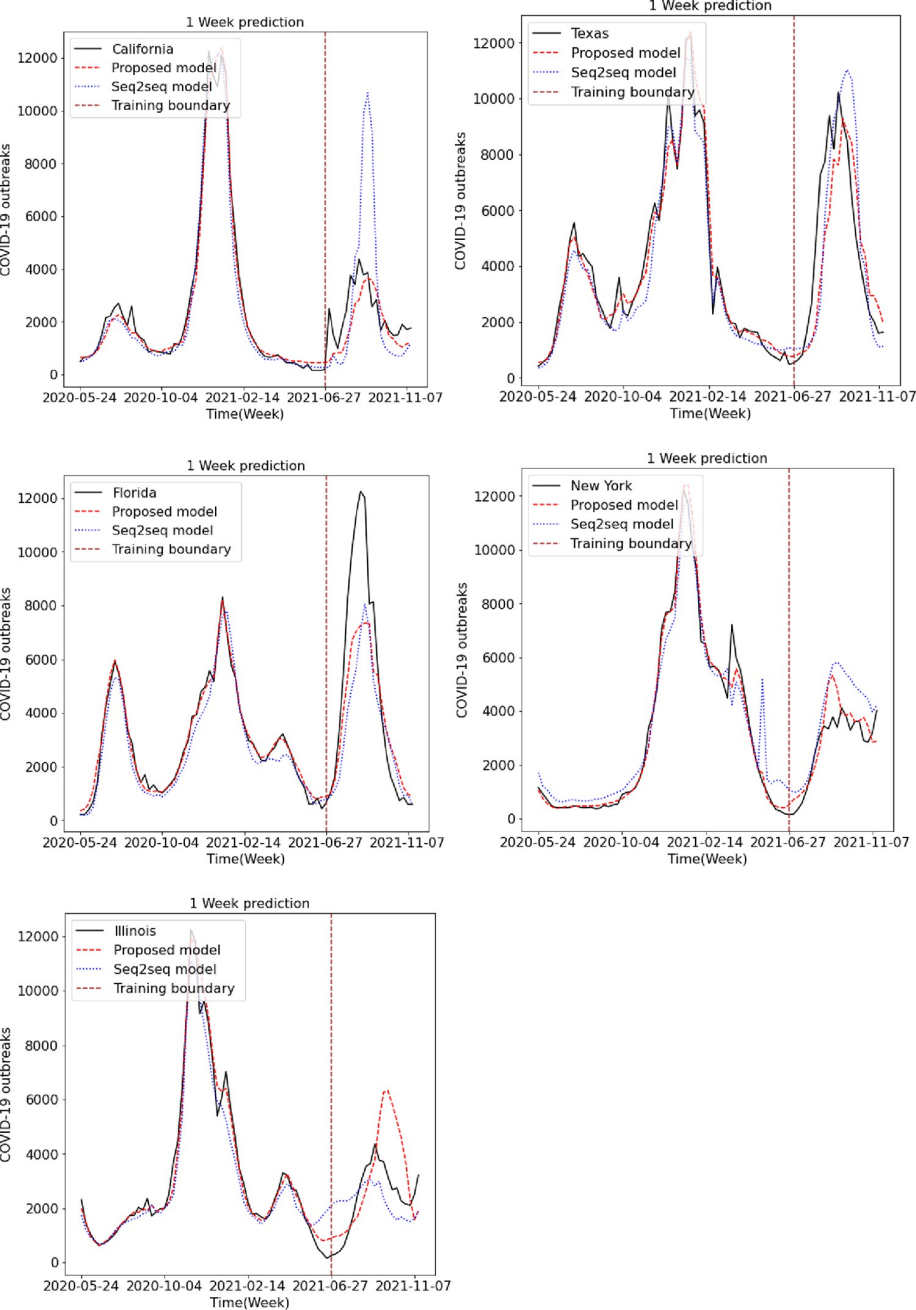
Week 1 prediction using LSTM model.

**Fig 10:**
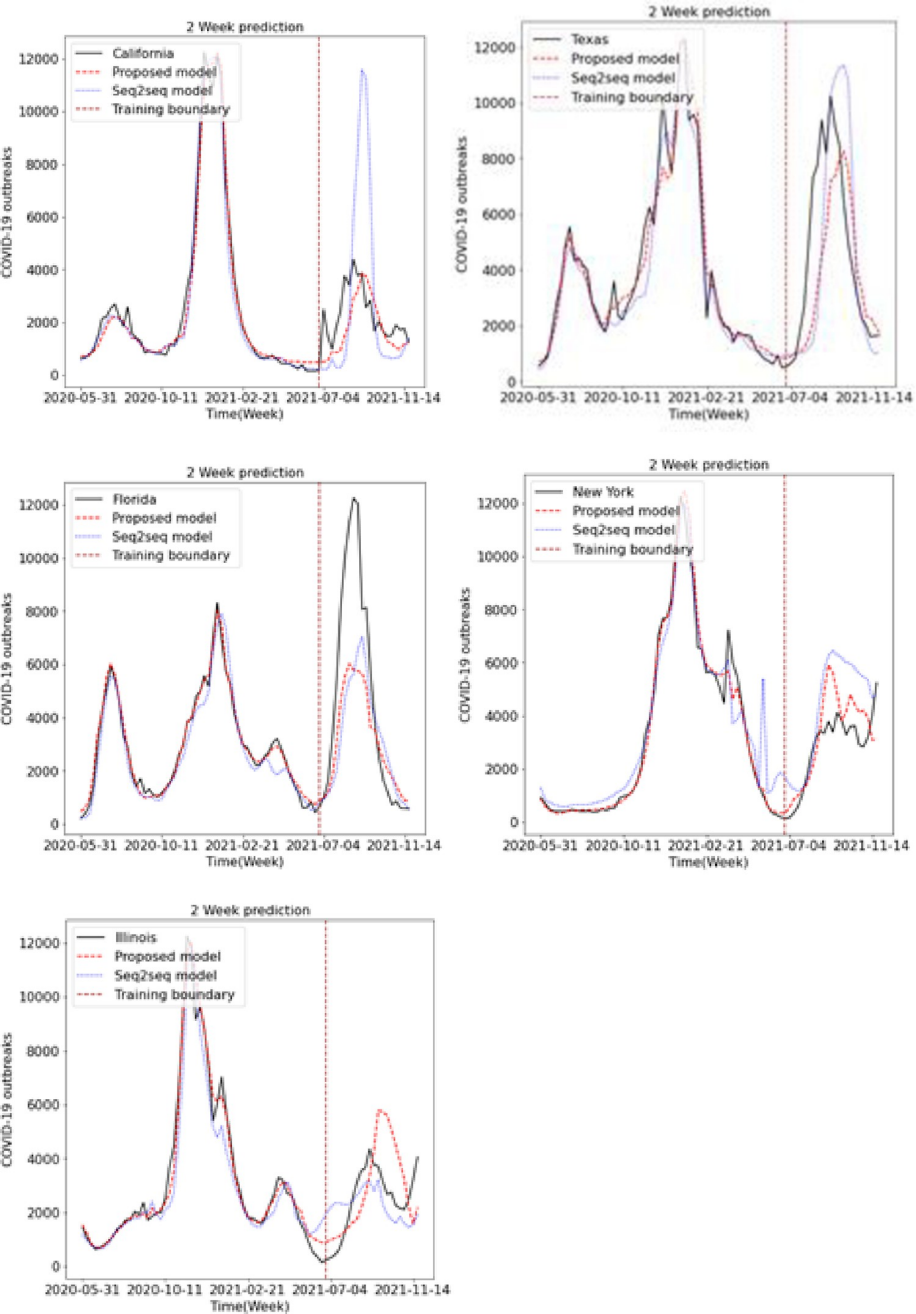
Week 2 prediction using LSTM model.

**Fig 11:**
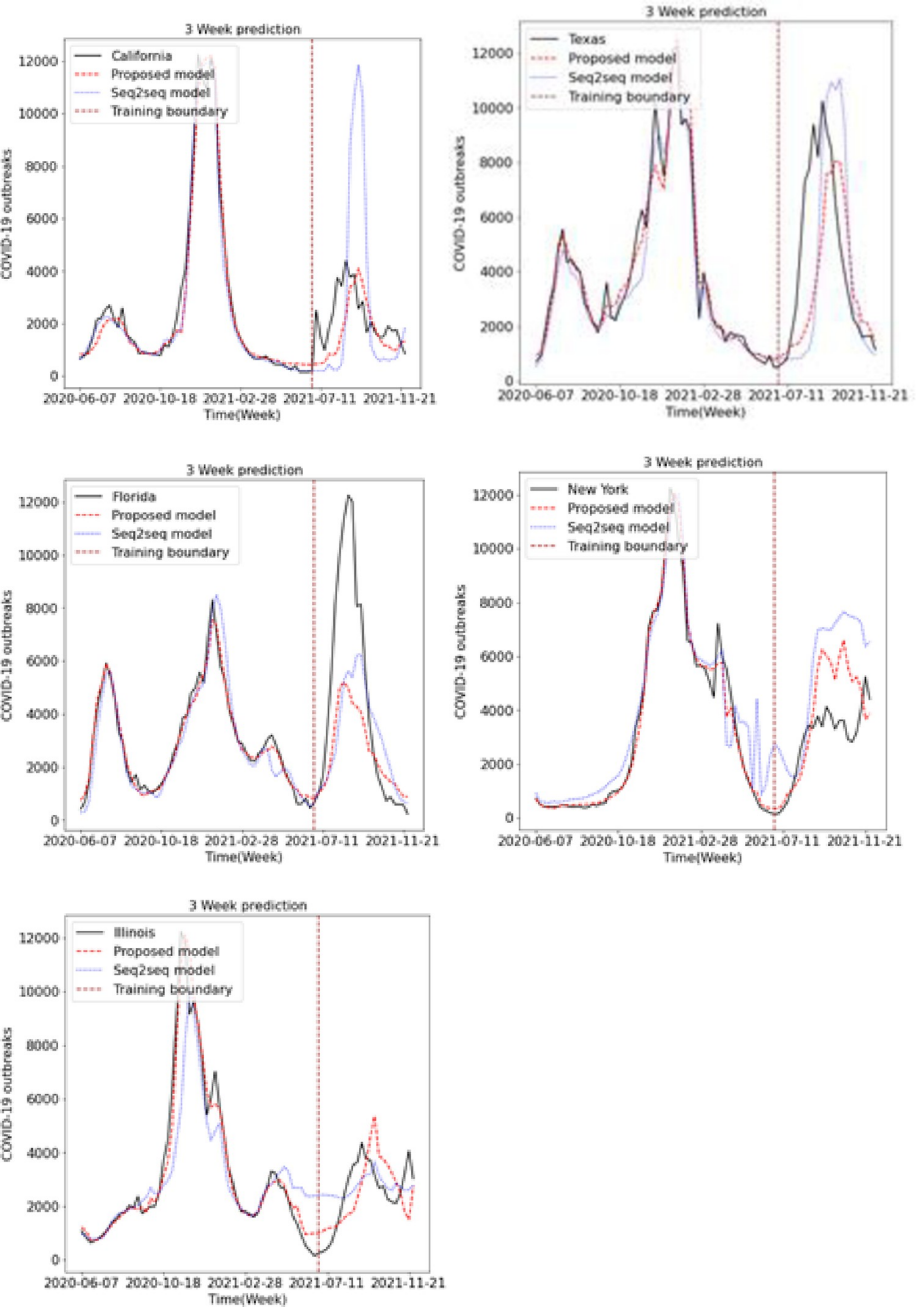
Week 3 prediction using LSTM model.

**Fig 12:**
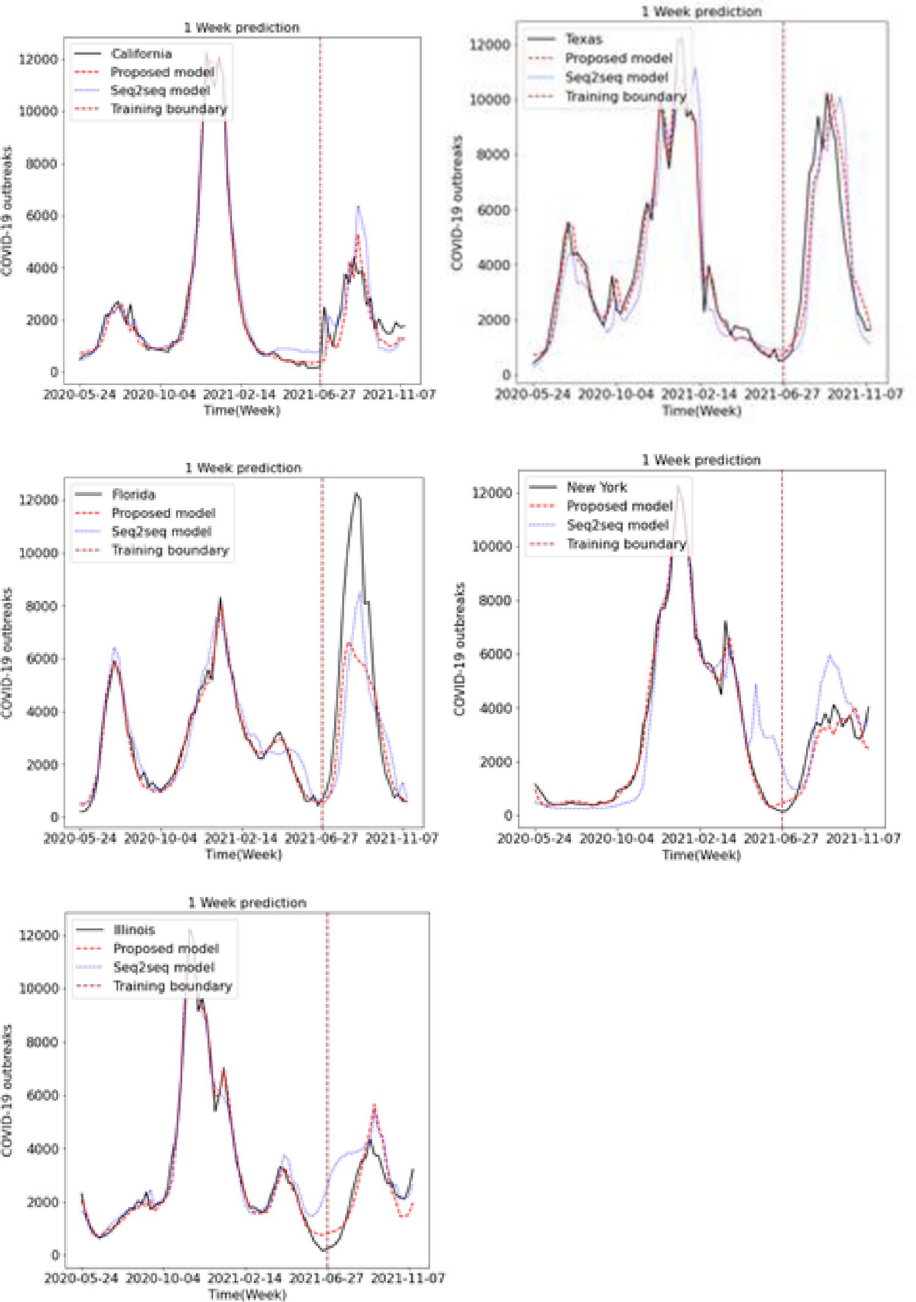
Week 1 prediction using Seq2Seq model.

**Fig 13:**
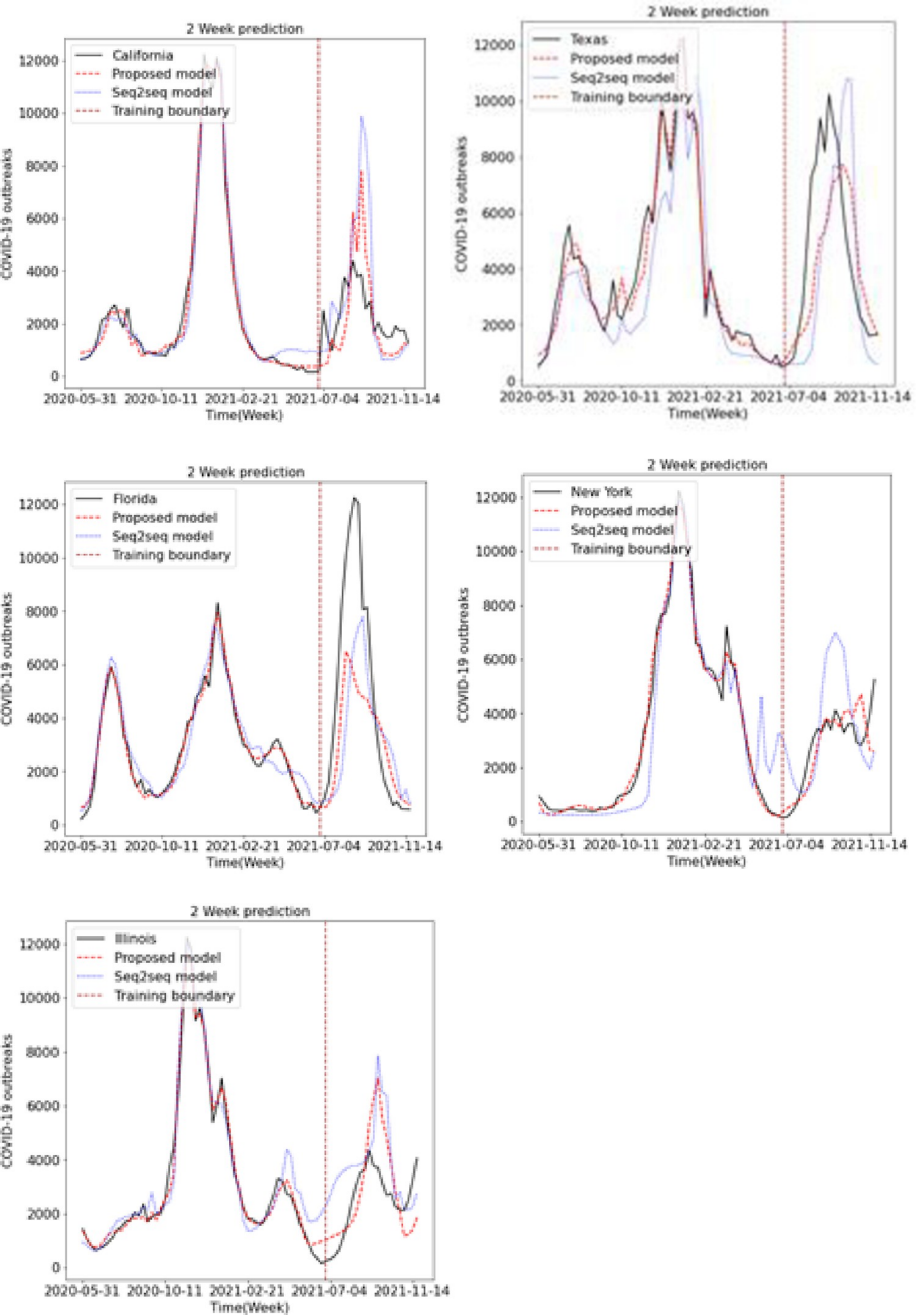
Week 2 prediction using Seq2Seq model.

**Fig 14:**
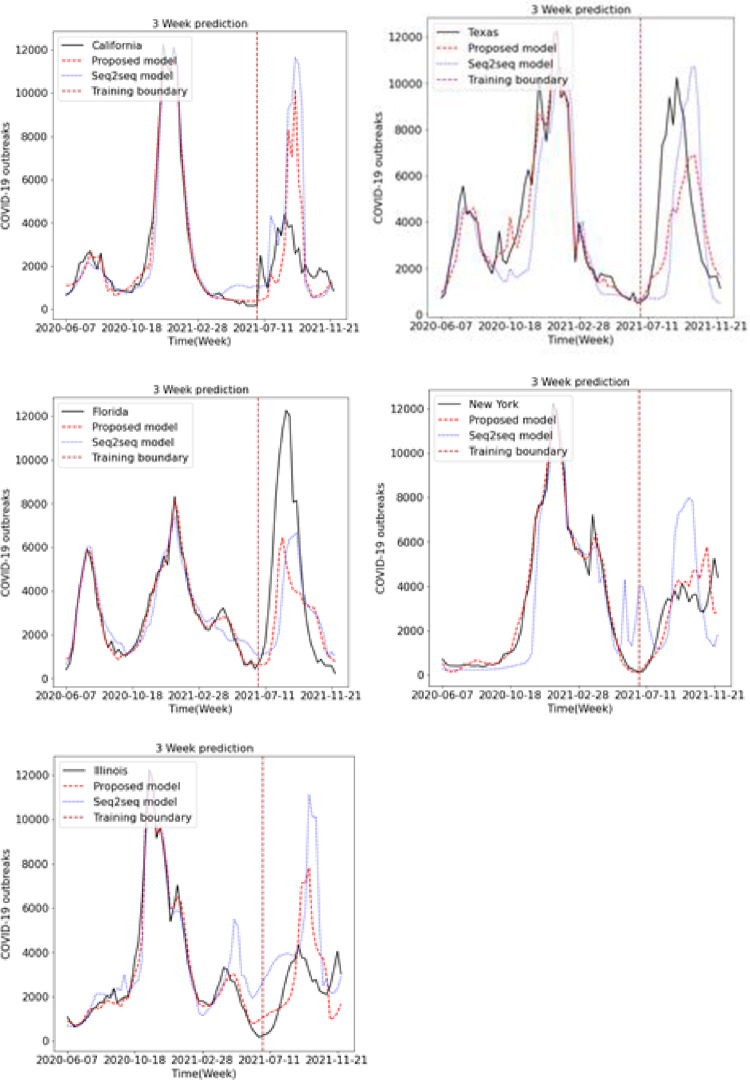
Week 3 prediction using Seq2Seq model.

We predicted the occurrence of COVID-19 using the existing LSTM and Seq2Seq models and the proposed LSTM and Seq2Seq models. Figs 9, 10 and 11 show the results of predicting COVID-19 from the 1st to the 3rd weeks in the five US states using the existing and proposed LSTM models. In each figure, the brown vertical dotted line indicates the period of the training data, the left part of the brown dotted line represents the training data, and the right part represents the test data. Looking at the predictions of the model from the test data on the right, we can see that the proposed model generally performs better in terms of accuracy with the actual COVID-19 outbreak data compared to the existing LSTM model. Figs 12, 13 and 14 show the results of predicting COVID-19 from the 1st to the 3rd week in the five US states using the existing and proposed Seq2Seq models. Similarly, in Figs 12, 13 and 14, the left side of the orange dotted line is the part of the learning data, and the right side is the part where the model predicts the test data. The prediction results of the Seq2Seq model also confirm that the proposed model predicts the COVID-19 outbreak data more accurately than the existing model, and the longer the parking time, the larger the error between the actual data and the prediction results. Table 4 shows the Pearson correlation coefficient and RMSE measurements for the predicted test data using both LSTM models. In most predictions, the Pearson correlation coefficient of the proposed model was higher than that of the existing LSTM model, and the RMSE of the proposed method was generally smaller than that of the existing LSTM model, indicating a relatively small error. Table 5 shows the Pearson correlation coefficient and RMSE for the predicted results using the existing and proposed Seq2Seq models. Similarly, in Table 5, the Pearson correlation coefficient is higher in most instances in the proposed model than in the previous model, and the RMSE is lower than in the prediction results of the existing model.

**Table 4:** LSTM model test set prediction accuracy evaluation.

States name	Prediction method	1-week prediction	2-week prediction	3-week prediction
		(PCC, RMSE)	(PCC, RMSE)	(PCC, RMSE)
California	LSTM model	0.69, 0.22	0.61, 0.26	0.45, 0.29
	Proposed model	**0.73, 0.07**	**0.65, 0.08**	**0.53, 0.1**
Texas	LSTM model	0.85, 0.17	0.71, 0.24	0.56, 0.28
	Proposed model	0.85, **0.14**	**0.72, 0.19**	**0.58, 0.23**
Florida	LSTM model	0.92, 0.22	0.86, 0.24	0.79, 0.26
	Proposed model	**0.96, 0.18**	**0.96, 0.23**	**0.96**, 0.27
New York	LSTM model	**0.94**, 0.11	**0.85**, 0.15	**0.76**, 0.24
	Proposed model	0.88, **0.06**	0.81, **0.09**	0.79, **0.13**
Illinois	LSTM model	0.33, **0.1**	0.36, **0.1**	0.57, **0.09**
	Proposed model	**0.65, 0.13**	**0.53, 0.12**	**0.53, 0.1**
Average	LSTM model	0.74, 0.16	0.67, 0.19	0.62, 0.23
	Proposed model	**0.81, 0.11**	**0.73, 0.14**	**0.67, 0.16**

**Table 5:** Seq2Seq model test set prediction accuracy evaluation.

States name	Prediction method	1-week prediction	2-week prediction	3-week prediction
		(PCC, RMSE)	(PCC, RMSE)	(PCC, RMSE)
California	Seq2Seq model	0.75, 0.09	0.67, 0.19	0.54, 0.3
	Proposed model	**0.81, 0.07**	**0.76, 0.11**	0.53, **0.2**
Texas	Seq2Seq model	0.85, 0.16	0.55, 0.28	0.44, 0.31
	Proposed model	**0.91, 0.11**	**0.71, 0.19**	**0.53, 0.24**
Florida	Seq2Seq model	0.91, **0.21**	0.79, 0.25	0.68, 0.29
	Proposed model	**0.94**, 0.22	**0.90, 0.24**	**0.83, 0.27**
New York	Seq2Seq model	0.83, 0.1	0.5, 0.16	0.21, 0.22
	Proposed model	**0.88, 0.05**	**0.78, 0.08**	**0.75, 0.09**
Illinois	Seq2Seq model	0.44, 0.13	0.42, 0.16	0.21, 0.26
	Proposed model	**0.86, 0.06**	**0.66, 0.12**	**0.49, 0.16**
Average	Seq2Seq model	0.75, 0.13	0.58, 0.20	0.41, 0.27
	Proposed model	**0.88, 0.10**	**0.76, 0.14**	**0.62, 0.19**

## Conclusion

In this study, we predicted COVID-19 outbreaks in five states (California, Texas, Florida, New York, Illinois) in the United States using the existing and proposed prediction methods based on LSTM and Seq2Seq+Attention models. The method proposed in this paper extracts 50 words associated with “covid19” from the pre-learned Word2Vec model, collects Google Trends data for that word, and compares the actual COVID-19 and Google Trends data to select the appropriate data for learning. Furthermore, it is a method of predicting the occurrence of COVID-19 by training the LSTM and Seq2Seq+Attention models, and weighting the results predicted by each model with the loss value for the verification data. The proposed method showed better performance and lower errors than the existing LSTM and Seq2Seq+ Attention models. However, to extract more appropriate keywords from news data using the Word2Vec model, it is necessary to extract keywords more precisely by considering the form or characteristics of the words.
